# Re‐framing Palliative Care for Latino Caregivers of Persons with Dementia

**DOI:** 10.1002/alz70858_097853

**Published:** 2025-12-24

**Authors:** Leah V Estrada, Sasha Perez, Nathan Goldstein, Albert Siu, Jennifer Reckrey

**Affiliations:** ^1^ Icahn School of Medicine at Mount Sinai, New York, NY, USA; ^2^ Dartmouth Health, Hanover, NH, USA; ^3^ Dartmouth University Geisel School of Medicine, Hanover, NH, USA; ^4^ Icahn School of Medicine, Mount Sinai Hospital, New York, NY, USA; ^5^ New York University Langone Health, New York, NY, USA

## Abstract

**Background:**

Latino older adults are 1.5 times more likely than non‐Latino White older adults to develop dementia. Palliative care is health care for individuals with serious illness and their families, focused on improving quality of life.^1^ The integration of palliative care and dementia care for persons with dementia (PWD) has been shown to improve symptom management (e.g., pain, agitation),^2,3^ and caregiver satisfaction.^2^ Despite known benefits, most PWD do not appear to receive palliative care.^4^ Therefore, we aimed to explore the palliative care needs of Latino PWD and their caregivers to see how to identify culturally relevant ways to deliver these services.

**Methods:**

In this qualitative study, semi‐structured, bilingual interviews were conducted in‐person, telephone, and on Zoom from June to November 2024 in New York City. After obtaining sociodemographic data about participants, we asked open‐ended questions exploring experience with dementia caregiving, navigating the healthcare system, and the role of culture. Interviews were recorded, transcribed, and analyzed using thematic analysis.

**Result:**

19 Latino family caregivers of PWD participated. Despite the focus on palliative care needs, none of the participants were familiar with palliative care. The challenges expressed by family caregivers were identified as: 1) personal challenges; 2) caregiver health challenges; 3) dementia care management; and 4) dementia symptoms. Participants did not identify with traditional, palliative care caregiver‐associated terminology (e.g., burden). There was a hesitancy to describe challenges as negative and instead that “things were under control”. The Latino cultural values of *familismo*, *dignidad*, and *respeto* mediated these challenges, offering an unique perspective on understanding the Latino dementia caregiver experience.

**Conclusion:**

To better support Latino family caregivers of PWD, palliative care teams should take into consideration Latino cultural values when tailoring care. Palliative care should be introduced as a culturally tailored set of services to assist families in ways that align with their values and caregiving practices. This reframing will help ensure that Latino caregivers view palliative care as a relevant and supportive resource.

